# Metabolic and cardiovascular responses to continuous and intermittent plank exercises

**DOI:** 10.1186/s13102-022-00613-z

**Published:** 2023-01-02

**Authors:** Zihao Huang, Biru Wang, Kangping Song, Shaoping Wu, Huimin Kong, Lan Guo, Qi Liang

**Affiliations:** 1 Department of Rehabilitation Medicine, The First Affiliated Hospital, Sun Yat-sen University, Guangzhou, Guangdong China; 2 Department of Rehabilitation Medicine, The Sixth Affiliated Hospital, Sun Yat-sen University, Guangzhou, China; 3 Rehabilitation Medicine Center, West China Hospital, Sichuan University, Chengdu, China; 4 Division of Cardiac Rehabilitation, Department of Physical Medicine and Rehabilitation, Xiangya Hospital of Central South University, Changsha, China; 5 Laboratory of Biomaterials and Translational Medicine, Center for Nanomedicine and Biotherapy Center, The Third Affiliated Hospital, Sun Yat-sen University, Guangzhou, China; 6 Guangdong Provincial People’s Hospital, Guangzhou, China

**Keywords:** Cardiorespiratory fitness, Anaerobic threshold, Resistance training, Muscle fatigue

## Abstract

**Background:**

Plank exercise (PE) is a whole-body isometric muscle training which is beneficial for physical health. However, none of the previous studies investigated the responses within a typical isometric muscle training or PE protocol consisting of multiple sets. The application of PE was restricted for the understudied metabolic and cardiovascular responses, especially for the patients with cardiovascular diseases. This study is to alleviate the safety concerns of PE by investigating the PE-induced metabolic and cardiovascular responses.

**Methods:**

Eleven male recreational-level college students completed a baseline cardiopulmonary exercise test, continuous PE (CPE) and intermittent PE (IPE). Ratio of maximal oxygen uptake per kilogram of body mass (%VO_2max_/kg), ratio of maximal heart rate (%HR_max_), and respiratory exchange ratio (RER) were continuously measured during PEs and divided into seven equal timepoints. Blood pressure (BP) was measured every minute during, before, and after PEs. A mixed-model repeated measures ANOVA was used to examine the interaction effect of exercise × phase.

**Results:**

The %VO_2max_/kg (F_6,69_=11.25, P < 0.001), %HR_max_ (F_6,65_=7.74, P < 0.001), RER (F_6,69_=11.56, P < 0.001), and BP (systolic BP, F_2,26_=8.42, P = 0.002; diastolic BP, F_2,24_=22.63, P < 0.001) increased by safe magnitudes. Compared with the corresponding period in the IPE group, the %VO_2max_/kg (33.5 [2.2] vs. 27.7 [1.9], P = 0.043) and %HR_max_ (63.2 [3.9] vs. 53.3 [2.1], P = 0.019) increased more significantly from the 40% duration of CPE. Systolic BP increased by larger magnitudes during CPE than IPE (154.2 [3.8] vs. 142.3 [4.8] mmHg, P = 0.002). RERs were over 1 during PEs without cardiovascular and metabolic variables over the anaerobic threshold.

**Conclusion:**

Energy was mainly supplied by anaerobic metabolism during PEs. CPE may be preferable for trainees aiming at anaerobic capacity enhancement. IPEs may be preferable to CPEs for youth patients with mild and borderline cardiovascular diseases due to their lower metabolic and cardiovascular responses.

## Background

Isometric exercises (IEs) are characterized by increased muscle tension without alteration in muscle length during contraction [1, 2]. They are widely applied in all-age training, sport injury prevention, and rehabilitation, as they help to promote muscle torque, muscle mass, and joint angle-specific stability [1, 3]. Over the past decade, IEs have been included in the exercise prescriptions of patients with cardiovascular diseases (CVDs) due to the associated benefits; these include improving cardiovascular and metabolic adaptations and lipoprotein profiles, and increasing insulin sensitivity [2, 4]. Plank exercises (PEs), IEs involving whole-body muscle contraction, are popular exercises that require the participants to keep a prone plank position while supported by the elbows and feet [5]. They are easily performed with various progressions, spine-friendly, and help to enhance core muscle stabilization and prevent sport injury [5]. In this regard, IEs and PEs are worth investigating.

Due to concerns regarding adverse events caused by excessive cardiovascular stress, e.g., blood pressure (BP), the application of IEs and PEs has been constrained despite the strengths and benefits [2, 6]. Over 330 million people were diagnosed with CVDs in China, and over 245 million were hypertensive [7]. Only small-muscle (handgrip), short-duration (2 min per set), and intermittent IEs were recommended in the hypertensive population [4]. IEs in larger muscles and with longer durations were considered to exhibit a more pronounced increase in cardiovascular and metabolic stress [6], which may be another cause of the limited application of large-muscle IEs or the lack of studies in which IEs were continuously performed to exhaustion. As the repetition maximum in isotonic exercise, the maximum duration of IE is essential to evaluate the relative intensity and exercise volume for prescribing effective and individualized training programs [8].

However, conflicting results were reported in previous studies that explored the metabolic and cardiovascular aspects of large-muscle IEs by sustaining single sets of 1–3-min leg extension [9–11]. Chapman et al. and Iellamo et al. reported lower-magnitudes increase in BP and oxygen uptake (VO_2_) during 1-min IEs compared with isotonic exercises [9, 10], while a greater increase in BP during IE was reported by Arimoto et al. and Koba et al. [11, 12]. The reasons for these conflicting results may relate to the different workloads among the compared protocols. To date, none of the previous studies investigated a typical exercise protocol with multiple sets [6].

As mentioned, PEs are popular IEs with various benefits involving whole-body muscles, including large muscles, often performed continuously or in multiple sets. There are few studies regarding the metabolic and cardiovascular response in PEs, and additional research is required. IEs in the trunk and lower extremities increased systolic BP (SBP), diastolic BP (DBP), heart rate (HR), and VO_2_ [2, 12–14], whereas previous studies mainly focused on comparing IEs with isotonic exercises; few focused on comparing a constant IE workload among different durations and protocols [6]. Consequently, these studies could not illustrate that applying large-muscle IEs to patients with CVDs was safe or provide a safe duration for performing large-muscle IEs. By investigating PEs, we found that the workload tended to be constant, the maximum duration was more easily measured, and dividing PEs into multiple sets was in accordance with the typical protocol.

Therefore, this study specific in large-muscle IEs was performed to measure and compare the acute metabolic and cardiovascular responses during two PE protocols, continuous (CPE) and intermittent PE (IPE). This study was also performed to alleviate concerns when prescribing PEs to patients with borderline or mild CVDs. We hypothesized that PEs could transiently increase safe-magnitude metabolic and cardiovascular responses, and the induced stress would be greater during CPEs.

## Methods

### Subjects

All subjects (mean [M] ± standard deviation [SD]; age, 21.1 ± 1.2 years; height, 1.73 ± 0.07 m; weight, 64.1 ± 8.6 kg; total plank duration, 336 ± 109 s) were recreational-level males who performed moderate-to-high intensity exercises 1–3 times a week for at least 1 year without integrating PEs into their training schedules. All subjects were required to abstain from tea, coffee, creatine supplementation, and strenuous exercise for 24 h before each of the three study visits. All subjects were familiar with the study procedure and provided written, informed consent prior to baseline measurements.

The inclusion criteria were: an age of 18–24 years, body mass index (BMI) in the normal range, answering “No” to all questions in the Physical Activity Readiness Questionnaire, and being able to maintain the plank position for more than 3 min. Subjects were excluded if they were diagnosed with known CVDs (e.g., hypertension or diabetes), had physical injuries that may affect PEs, or were undergoing nutritional programs for weight loss or muscle gain.

### Study design

This observational study compared the induced cardiovascular and metabolic stress when performing two PEs. A sample of 11 recreational-level, collegiate male subjects were recruited to attend three laboratory visits, including a baseline measurement and two PE sessions. The study design and all procedures were approved by the ethics committee of The First Affiliated Hospital, Sun Yat-sen University [Number: 2019(036)]. Laboratory visits were performed in the Cardiac Rehabilitation Department of Guangdong Provincial People’s Hospital, People’s Republic of China, between May 2018 and April 2019.

### PEs

**Fig. 1:**
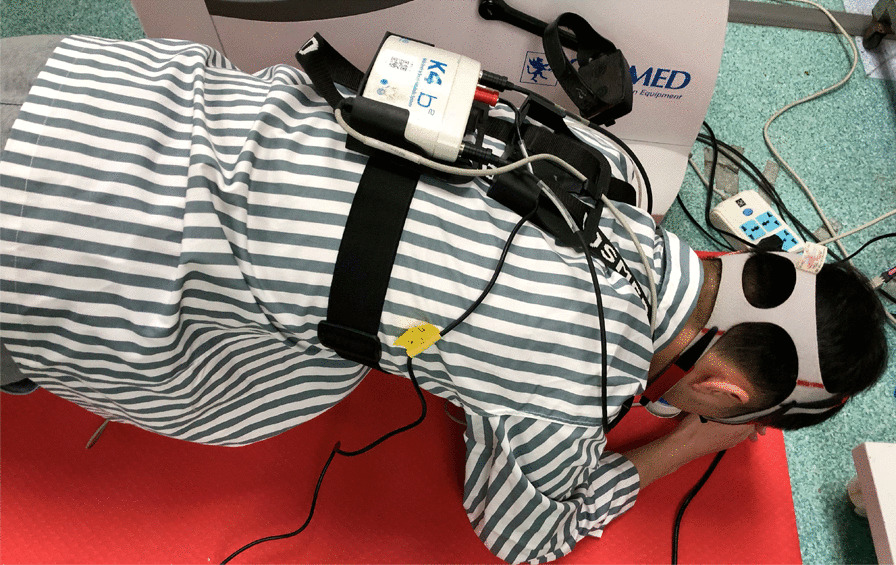
Demonstration of plank exercise and measurements

The PE protocols included a CPE and an IPE (Fig. 1). During the CPEs, subjects were required to perform the PE for as long as possible. During the IPE, refer to the large-muscle IE protocols designed by Wiles et al., Chapman et al. and Iellamo et al., subjects were required to complete three repetitions of a 1-min PE with a 1-min intermittent rest interval, taking 6 min in total [9, 10, 15]. A 2-week wash-up period was included between two consecutive study visits.

### Baseline measurements

Cycling-based cardiopulmonary exercise testing (CPET; K4b2; COSMED, Rome, Italy; and RS400; Polar Electro, Kempele, Finland) was performed by Bruce protocol after collecting the demographic information (age, height, weight, BMI). Oxygen uptake per kilogram of body mass (VO_2_/kg) and HR at the anaerobic threshold (VO_2AT_/kg, 20.9 ± 5.6 ml min^−1 ^kg^−1^; HR_AT_, 133 ± 10 bpm) and at maximal (VO_2max_/kg, 33.8 ± 4.6 ml min^−1 ^kg^−1^; HR_max_, 182 ± 14 bpm) were measured using CPET.

### Data collection

The metabolic response included blood lactate (La; Lactate Scout; EKF Diagnostics, Cardiff, United Kingdom)—measured before and after PEs—continuous RER, %VO_2max_/kg and energy expenditure (EE). EE was calculated using previously reported formulas [16]. Blood samples were collected from the tip of the middle finger after disinfection. Cardiovascular responses included continuous %HR_max_ (Polar Electro) and noninvasive BP (Tango M2, USA). SBP and DBP were measured every 1 min during PEs, as well as before and after exercise; %HR_max_ and %VO_2max_/kg were calculated using the HR and VO_2_/kg proportions of HR_max_ and VO_2max_/kg, respectively.

### Statistical analysis

To reduce random errors, continuous data in CPE, including %VO_2max_/kg, RER, EE, and %HR_max_, during PEs, were divided into seven timepoints with equal intervals (rest; 20, 40, 60, 80, and 100% of the duration; and recovery). In IPE, continuous data in the 1-min training session were classified into the 20%, 60% and 100% of the duration respectively, and the data in the 1-min rest interval were classified into the 40%, 80% of the duration and the recovery respectively. The means of the continuous data were individually calculated within each timepoint before statistical analysis. Due to the different CPE duration among the subjects, BP was analyzed by three timepoints, rest, exercise, and recovery. The exercise BP was presented by the mean of all BP measured during exercise. All data are presented as M and SD.

A mixed-model repeated measures analysis of variance was used to examine the interaction effect of exercise × phase (2 × 2 for La, 2 × 3 for BP, and 2 × 7 for %VO_2max_/kg, RER, EE, and %HR_max_), as well as the follow-up simple effects of phase and exercise (CPE and IPE). Bonferroni adjustment was used for pairwise comparison. All statistical analyses were performed using IBM SPSS Statistics for Windows, Version 23.0 (Armonk, NY: IBM Corp.), and statistical significance was set at P < 0.05. Sample size was calculated by PASS 2021 using repeated measures analysis design, setting a minimum power at 0.8, α at 0.05, a between level at 2 and a within level at 7. According to the calculation report, twenty-two subjects, two groups with 11 subjects of each, were required. Due to the crossover design in this study, eleven subjects satisfied the statistical power.

## Results

### Metabolic responses

**Fig. 2:**
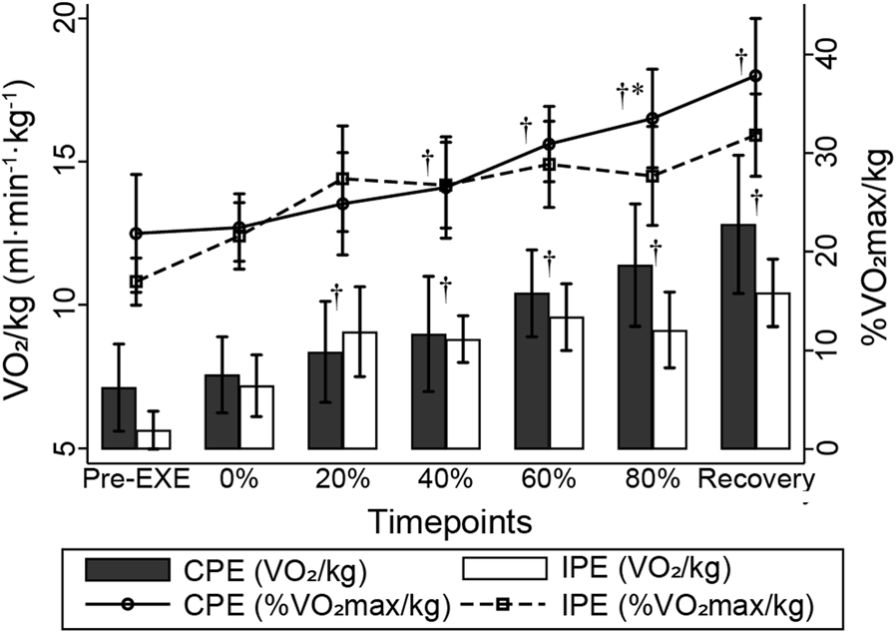
VO_2_/kg and %VO_2max_/kg during plank exercises, [Mean (Standard error)]. *Pre-EXE* pre-exercise period; *P < 0.05 in multiple comparison in simple effect of exercise, CPE versus IPE; ^†^P < 0.05 in multiple comparison in simple effect of phase versus reference Pre-EXE value

Significant differences were observed in the effects of phase (F_6,69_=11.25, P < 0.001) and exercise (F_1,69_=4.57, P = 0.036) for %VO_2max_/kg (Fig. 2). The %VO_2max_/kg (standard mean difference [SMD], 95% confidence interval [CI] [0.19–11.46], P = 0.043) at 80–100% of the CPE duration was significantly higher than during the third session of IPE. Differences were not statistically significant for pre-exercise %VO_2max_/kg until 40% of the CPE duration until recovery (P < 0.01). In the IPE, a significant increase in %VO_2max_/kg was observed from the second PE session (P < 0.05). None of the values for %VO_2max_/kg during PEs were over the VO_2AT_/kg proportion of VO_2max_/kg (61.8%). No significant interaction effects were identified between exercise × phase in %VO_2max_/kg (P > 0.05).

**Fig. 3:**
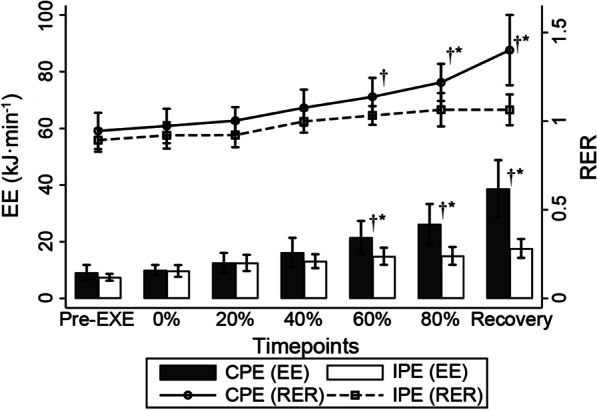
EE and RER during plank exercises, [Mean (Standard error)]. *Pre-EXE* pre-exercise period; *P < 0.05 in multiple comparison in simple effect of exercise, CPE versus IPE; ^†^P < 0.05 in multiple comparison in simple effect of phase versus reference Pre-EXE value

Significant interaction effects (F_6,69_ = 3.35, P = 0.006; Fig. 3), and simple effects of phase (F_6,69_ = 11.56, P < 0.001) and exercise (F_1,69_ = 34.08, P = 0.016) were identified in the RER. Differences were only significant for the pre-exercise RER from the 60% duration of CPE and the second rest period until recovery (P < 0.01). From these timepoints, RER increased to a higher level than in the IPE (SMD, 95% CI [0.04–0.27], P = 0.008) and increased to over 1.0 (CPE, 95% CI [1.03–1.24]; IPE, 95% CI [1.00–1.13]). Significant interaction effects (Fig. 3, F_6,65_ = 7.99, P < 0.001) of phase (F_6,65_ = 16.33, P < 0.001) and exercise (F_1,65_ = 40.37, P < 0.001) were identified in EE. A significantly higher increase in EE was observed from 60 to 80% of the CPE duration (SMD, 95% CI [1.50–11.74], P = 0.012). As with the RER, a similar tendency to increase was also observed in EE for the corresponding periods (P < 0.01).

A significant interaction effect was also identified in La (F_1,20_ = 13.56, P = 0.001; Table 1). Significant differences were observed in the effects of phase (F_1,20_ = 49.61, P < 0.001) and exercise (F_1,20_ = 11.75, P = 0.003); however, no significant differences were observed before exercise. La increased significantly after PEs (P ≤ 0.001), with a significantly higher magnitude observed in the CPE than IPE (SMD, 95% CI [0.38–1.58], P = 0.003).

**Table 1:** Blood pressure and lactate during PEs, Mean (SD)

Variables	Phase	CPE	IPE	F_interaction_	F_exercise_	F_phase_
SBP	Pre-EXE	126 (10)	130 (13)	2.78	0.42	8.42*
	Exercise	154 (15)	141 (14)			
	Recovery	136 (13)	133 (17)			
DBP	Pre-EXE	76 (9)	77 (10)	1.54	1.46	22.63^†^
	Exercise	95 (14)	82 (11)			
	Recovery	73 (12)	71 (11)			
La	Pre-EXE	1.8 (0.6)	2.0 (1.0)	13.56^†^	11.75*	49.61^†^
	Recovery	5.0 (1.6)	2.8 (0.6)			

*Pre-EXE* pre-exercise period, *SBP* systolic blood pressure, *DBP* diastolic blood pressure, *La* blood lactate, *CPE* continuous plank exercise, *IPE* intermittent plank exercise. F_interaction_, F value of interaction effect of phase × exercise by repeated measures analyses of variance (RMANOVA); F_exercise_, F value of simple effect of exercise by RMANOVA; F_phase_, F value of simple effect of phase by RMANOVA

*P < 0.01, ^†^P < 0.001

### Cardiovascular responses

**Fig. 4:**
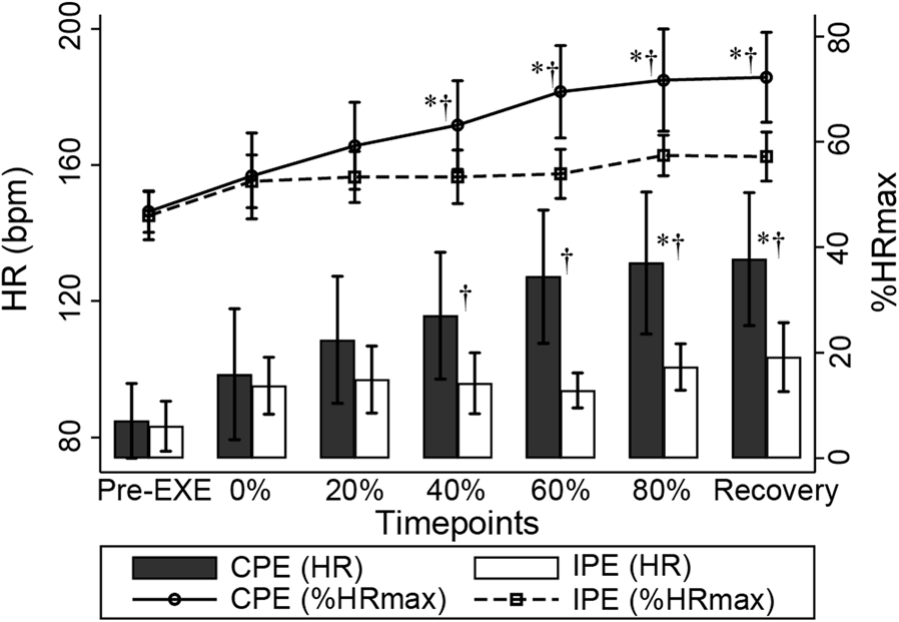
HR and %HRmax during plank exercises, [Mean (Standard error)]. *Pre-EXE* pre-exercise period; *P < 0.05 in multiple comparison in simple effect of exercise, CPE versus IPE; ^†^P < 0.05 in multiple comparison in simple effect of phase versus reference Pre-EXE value

Significant interaction effects were identified in %HR_max_ (Fig. 4, F_6,65_ = 2.73, P = 0.02), and significant differences were observed in the effects of phase (F_6,65_ = 7.74, P < 0.001) and exercise (F_1,65_ = 32.30, P < 0.001). The %HR_max_ increased significantly from 40% of the duration of the CPE and the second session of the IPE to the recovery compared with the pre-exercise level (P < 0.01). The %HR_max_ for the CPE increased by a significantly higher magnitude from 40% of the CPE duration than the second session of IPE (SMD, 95% CI [1.65–18.17], P = 0.019). None of the values for %HR_max_ during the PEs were over the HR_AT_ proportion of HR_max_ (73.1%).

Nonsignificant interactions or simple exercise effects were identified for BP (P > 0.05; Table 1), and a significant difference was observed in the simple effects of phase (SBP, F_2,26_ = 8.42, P = 0.002; DBP, F_2,24_ = 22.63, P < 0.001). SBP (SMD, 95% CI [6.5–30.5], P = 0.002) and DBP (SMD, 95% CI [4.0–19.1], P = 0.002) increased during PEs, and SBP returned to the pre-exercise level during recovery (P > 0.05); however, a significant decrease to a lower level than observed pre-exercise was observed in DBP (SMD, 95% CI [–16.9 to –1.3], P = 0.018).

## Discussion

In this exploratory analysis, PEs evoked significant safe-magnitude metabolic and cardiovascular responses; the stress may be greater in CPE. The responses were not significant until 40% of the CPE duration or the second session of IPE. Additionally, La increased, and RERs were over 1; neither the %VO_2max_/kg nor %HR_max_ exceeded the %VO_2AT_/kg or %HR_AT_, respectively.

Prior research suggests that HR, VO_2_/kg, and BP increase during lower-extremity IEs [17, 18]. While similar results were observed in the presented study, the magnitudes of VO_2_/kg were much higher [10, 13]. The reason for the larger metabolic response may be that compared with previously investigated exercises, PEs require higher levels of neuromuscular activation to meet the increasing demands of muscle oxygenation and energy supply [17]. The presented results of EE were also consistent with this statement. Increased HR and BP during small- and large-muscle IEs have been widely reported [12–14, 19] due to the increased cardiac output, circulating norepinephrine, and metaboreflex activation [6]. The magnitudes of HR and BP elevation were similar to those reported in previous studies of small- and large-muscle IEs [12–14][19], which may strengthen the statement that cardiovascular responses to IEs were irrespective of the types and mass of muscle contraction due to the decreased cardiac preload and increased afterload [6, 20].

Both responses were greater in CPE, and similar results were reported in studies comparing IEs and isotonic exercises [6, 14]. Larger-magnitude increase in CPE may be caused by a longer duration of Valsalva-like physiological changes and autonomic nervous activation. During IEs, the Valsalva maneuver is easily performed and the PE-induced responses are similar to performing Valsalva maneuver for 15–20 s; intrathoracic pressure (ITP) and systemic vascular resistance (SVR) increase, followed by a decrease in venous return and stroke volume [6, 14, 21]. To maintain the cardiac output for exercise, HR was proportionally increased in compensation [6, 14]. These physiological changes may take minimum 30 s to reach a steady state after the onset of SVR strain and increased ITP [21]. For the PE is not a Valsalva maneuver, the time to steady state may be not 30 s and will be discussed in the following part. Steady state may be achievable for CPE, but not IPE, which demands 1-min exercise followed by rest. Moreover, higher exercise volume in may have led to longer exposure to circulating norepinephrine and metaboreflex activation [6]. Apart from these, the IPE allows for greater muscle reperfusion, [6] which may mitigate the Valsalva-like effects of CPE. The lower DBP observed during recovery in the present study was also reported during limb remote ischemic conditioning [22]. This may be attributed to the regulation of the autonomic nervous system and increased secretion of vasodilatory substances (e.g., nitric oxide) amongst distal limbs[22]. Another reason may be a sudden drop of central venous pressure and expansion of the superior and inferior vena cavae induced by the decreased ITP [21]. These physiological changes result in a decrease of the peripheral venous pressure and DBP [21, 22]. However, the underlying mechanisms remain understudied. Nevertheless, neither the HR nor BP during PEs exceeded the exercise-termination limits, suggesting a wider application of PEs in youth patients with borderline or mild CVDs.

In our study, differences in %HR_max_ were nonsignificant compared with the rest values until over 40% of the exercise duration of PEs. In the following exercise timepoints, the differences were not significant from the %HR_max_ at 40%. Combined with the total plank duration in our study, it suggests the time to steady state may around 1–2 min, and it was interrupted by the rest intervals in IPE. The phase effects of %VO_2max_/kg was consistent with those in %HR_max_, but the time was delayed in RER and EE. These results were not reported by prior studies. During the first 1–2 min of exercise, energy was mainly supplied by adenosine triphosphate-phosphocreatine and glycolysis, and prolonged exercises relies on aerobic metabolism, characterized by evident increased in VO_2_/kg and RER [23]. In this case, the causes of the advance steady state of VO_2_/kg may be the response of sudden raise in norepinephrine secretion and restricted right ventricular output [6, 21]. For RER was a parameter describing the pathways of energy supplied, the time to steady state in this study was also consistent with the energy metabolism sequence and features [23]. These cardiovascular and metabolic features were studied based on the constant workload exercises, and the similar results in this study also implied PE is a constant workload exercise [23, 24]. [25] Based on the features of constant workload exercises, a light workload may evoke a slighter increase in HR and VO_2_/kg, and the variables are maintained at steady states until exercise termination after minutes of increase [24]. In this regard, combining the concerns of time effects and the implied physiological changes, cardiovascular stress may be higher if performing a prolong PE. The IPE may be more friendly for youth patients with borderline or mild CVDs with similar training-induced responses, and the extended exercise duration or augmented sets could be individualized.

Interestingly, none of the %VO_2max_/kg and %HR_max_ over the %VO_2AT_/kg and %HR_AT_ accompanied by RER over 1 with increased La. The reasons are still unexplained. The anaerobic threshold was measured by CPET, during which respiration depth, rate, HR, and stroke volume were increased to meet the incremental muscular oxidation [25]. Exercise over the anaerobic threshold (RER = 1) in CPET suggests that the energy supplied by oxidative system is inadequate to maintain the required activity level; thus, glycolysis is activated with La accumulation [25, 26]. La accumulation could decrease plasma pH, suppress muscle contraction, and facilitate peripheral and central fatigue [27]. Consequently, adjusted by the central nervous system, the expired CO_2_ concentration increases to maintain the blood plasma’s acid-base equilibrium [28].

Essentially, failure to maintain the PE may be caused by impeded respiration, oxygen delivered, and La clearance, leading to suppressed muscle contraction. PEs share the same muscles around the rib cage and abdomen [5, 29]; thus, identical to other IEs, PEs could increase the ITP [6]. The diaphragm must modulate the ITP for respiration when the diaphragm and abdominal muscles contract concurrently [30]. The range of motion in the rib cage was also restricted, and the flow-generating function of the diaphragm decreased [30]. As a result, the peak VO_2_/kg was affected [30]. Conversely, increased muscle pump and vasodilation are significant during CPET [2, 6]. However, during IEs, the muscles tend to impede capillary vasodilation, thereby increasing the SVR [2]. Muscle exercise-induced perfusion was impeded, leading to relative ischemia [2]. This phenomenon is also consistent with the higher exercise DBP, increased %VO_2max_/kg at CPE recovery, and rest intervals of IPE in our study. La could not be promptly removed from the muscles due to the impeded perfusion leading to decreased muscle contraction and exacerbated muscle fatigue [2, 26, 27]. La may accumulate within the contracted muscles and be removed during the IPE’s rest intervals, leading to a lower La than CPE. In this regard, energy may be mainly supplied by anaerobic metabolism during PEs, and enhanced lactate tolerance and skeletal muscle capillary density may prolong the plank duration.

Overall, there were some limitations in our study. First, only 11 subjects were included in our study as participants were required to perform at least 3 min of the PE. Nevertheless, previous studies in this field recruited 7–20 subjects, and the power had been checked was satisfactory by the prior and posteriori sample size calculations. Second, the exercise volumes were not equal for each participant; the responses to completing a 1-min PE session may be mild in subjects with longer CPE durations. The metabolic and cardiovascular responses may be less significant; however, the relationship between exercise intensity and plank duration is still unknown, and we wanted to guarantee that subjects could complete all three IPE sessions. Additionally, the IPE was selected to include rest intervals, similar to a typical training session. Third, whole-body muscle contraction was required, and noninvasive BP measurement was difficult under this circumstance. Although the random error may be greater, it should affect all subjects in both PEs in the same way. Finally, the effectiveness and safety of applying PE in patients with CVDs could not be entirely guaranteed only by our study, but this study could be a starting investigation in expanding the application of large-muscle IE to patients with CVDs. At the present stage, further studies in applying PEs to youth patients with borderline or mild CVDs and elder healthy population is worth exploring.

## Conclusion

A single bout of short-duration PE may not evoke significant metabolic and cardiovascular responses; however, significant in longer-duration or multiple boots of PE, and the responses are similar to constant workload exercise. During PE, regardless of protocols and PE duration, energy may be mainly supplied by glycolysis. CPE may be preferable in the healthy population aiming at anaerobic training for greater metabolic and cardiovascular responses with higher EE. IPE may be more practical and safer for youth patients with borderline or mild CVDs under instruction and monitoring.

## Data Availability

The datasets used and analysed during the current study are available from the corresponding author on reasonable request.

## Abbreviations

AT: Anaerobic threshold
BP: Blood pressure
BMI: Body mass index
CPET: Cardiopulmonary exercise testing
CVDs: Cardiovascular diseases
CI: Confidence interval
CPE: Continuous plank exercise
DBP: Diastolic blood pressure
EE: Energy expenditure
HR: Heart rate
HR_AT_: Heart rate at the anaerobic threshold
IPE: Intermittent plank exercise
ITP: Intrathoracic pressure
%VO_2max_/kg: Ratio of maximal oxygen uptake per kilogram of body mass
La: Lactate
M: Mean
max: Maximal
HR_max_: Maximal heart rate
VO_2max_/kg: Maximal oxygen uptake per kilogram of body mass
VO_2_: Oxygen uptake
VO_2_/kg: Oxygen uptake per kilogram of body mass
VO_2AT_/kg: Oxygen uptake per kilogram of body mass at the anaerobic threshold
PEs: Plank exercises
RER: Respiratory exchange ratio
SD: Standard deviation
SMD: Standard mean difference
